# Epidemiological, Genetic, and Phenotypic Characteristics of Non-Typhoidal *Salmonella* in Young Children, as Obtained from a Tertiary Hospital in Guangzhou, China

**DOI:** 10.3390/microorganisms11102433

**Published:** 2023-09-28

**Authors:** Baiyan Gong, Yulian Feng, Zhenxu Zhuo, Jingjie Song, Xiankai Chen, Xiaoyan Li

**Affiliations:** Clinical Laboratory, Fifth Affiliated Hospital, Southern Medical University, Guangzhou 510000, China; gong1078496950@outlook.com (B.G.);

**Keywords:** non-typhoidal *Salmonella*, serotype, subtype, infection rate, risk factors, resistance

## Abstract

Gastroenteritis caused by non-typhoidal *Salmonella* (NTS) is a significant disease in childhood, ranking as the seventh-leading cause of diarrhea mortality in children aged < 5 years. To understand the epidemiological, genetic, and phenotypic characteristics of NTS, 465 anal swabs from children aged < 5 years in a tertiary hospital in Conghua District, Guangzhou, China, were collected from June to October 2021. An average prevalence of 35.27% (164/465) was observed, with whole genome sequencing identifying 11 serotypes, among which *Salmonella 1,4,[5],12:i:-* was the most prevalent (65.24%, 107/164). Meanwhile, ST34 was found to be the predominant subtype. Children who are breastfed, eat fresh food, and have good hygiene habits show a relatively low prevalence of NTS. Fever is a common symptom that may be caused by NTS infection. Antimicrobial resistance testing revealed that the majority of strains were resistant to tetracycline (83.5%) and ampicillin (82.3%), with multi-drug resistance (MDR) observed in 50.61% (83/164) of all strains tested. The predominant resistance spectrum presents as tetracycline-ampicillin-chloramphenicol-trimethoprim-sulfamethoxazole (30.49%, 50/164). The antimicrobial resistance rates (2.4%, 9.8%, 9.8%, 10.4%, 9.1%, and 3.7%, respectively) of cephalosporins (cefepime, cefuroxime, cefuroxime axetil, ceftriaxone, ceftazidime, and cefoxitin) were low. Therefore, continued surveillance of the prevalence and MDR profiles of NTS, along with the rational use antibiotics, is required. This protocol is significant for preventing further dissemination of NTS and formulating effective prevention and control strategies.

## 1. Introduction

Non-typhoidal *Salmonella* (NTS) infection is a major international public health concern, and it adds an enormous financial burden to the healthcare system. According to the Global Burden of Disease Study 2016, NTS was the sixth-leading cause of diarrhea (64 million episodes, 95% uncertainty interval (UI) 31.32–122.03) in children younger than 5 years, and the seventh-leading etiology for diarrhea mortality (37,410 deaths, 95% UI 16,659–64,509) in this age group at the global level [1]. The severity of NTS infection depends on the host factors and the serotype of the infection [2]. In humans, NTS usually causes self-limiting gastroenteritis, presenting with diarrhea, abdominal pain, nausea, emesis, and fever. Sometimes, however, NTS can cause serious and potentially life-threatening infection in infants, the elderly, and patients with immune deficiency [3]. *Salmonella* is a major cause of global foodborne diseases, accounting for 80.3 million foodborne cases per year [4]. Reports of *Salmonella*-associated foodborne outbreaks are mainly associated with eggs, meat, poultry, and some fresh vegetables (e.g., tomato and cucumbers) [5,6,7,8,9,10]. In addition to foodborne NTS acquisition, humans can acquire *Salmonella* infection by ingesting contaminated water, through contact with animals, and by person-to-person transmission [11,12].

According to the White–Kauffmann–Le Minor scheme, over 2600 *Salmonella* serotypes have been identified worldwide, of which 292 different serotypes have been reported in China [13]. *S. Enteritidis* and *S. Typhimurium* are considered by the United States and European Food Safety Authority (EFSA) as the two most common serotypes causing human non-typhoidal salmonellosis [14,15]. *Salmonella 1,4,[5],12:i:-*, as a monophasic variant of *S. Typhimurium*, began to appear in the mid-1990s, and is currently one of the most common serotypes [16]. A previous study conducted in Guangxi, China, found that *S. Typhimurium*, *S. Enteritidis*, and *S*. *1,4,[5],12:i:-* were the most commonly identified serotypes among patients with diarrhea [17].

The emergence of multidrug-resistant (MDR—resistance to three or more different classes of antimicrobials) bacterial pathogens poses a serious threat to public health [18]. It is reported that the rate of MDR *Salmonella* increased to 40% in the last decade of the 20th century [19]. The ASSuT tetra- (ampicillin, streptomycin, sulfonamides, and tetracycline) and ACSSuT (ampicillin, chloramphenicol, streptomycin, sulfonamides, and tetracycline) penta-resistant patterns are the most frequently reported antibiotic resistance patterns in *S*. *Typhimurium* [20,21]. In recent years, fluoroquinolones, azithromycin, and cephalosporins have become the common choice for the clinical treatment of *Salmonella* infection [19]. However, over time, *Salmonella* strains resistant to these antibiotics have gradually begun to be identified in humans worldwide. Therefore, it is important to monitor and prevent the importation and spread of antibiotic-resistant *Salmonella*.

Whole genome sequencing (WGS) has been vital for identifying pathogens, understanding genomic epidemiology, tracing the source of outbreaks, and the rapid temporal and spatial evolution of antimicrobial resistance (AMR) in bacterial pathogens [22]. Multilocus sequence typing (MLST) is mainly applied to understand the hereditary backgrounds, describe the genetic relatedness, and trace the evolutionary paths of *Salmonella* subtypes (STs) [13]. In the current study, we characterized the prevalence, possible risk factors, and antibiotic resistance of NTS infection in pediatric patients (aged under 5 years) in a tertiary hospital in Conghua District, Guangzhou, China. Meanwhile, WGS was used to describe the genetic characteristics of the disease, including serotypes, STs, evolutionary relationships and antibiotic resistance genes (ARGs). There are numerous contraindications for the use of clinical drugs in children, posing significant challenges to clinical diagnosis and treatment. This study aims to identify the epidemiological characteristics of NTS, and the information provided will assist in developing a comprehensive national surveillance program to guide clinicians in selecting appropriate treatments.

## 2. Materials and Methods

### 2.1. Ethics Statement

Scientific approval and ethical clearance for this study was provided by the Ethics Committee of the Fifth Affiliated Hospital of Southern Medical University (2021-JYK-K-002). Prior to collection of anal swabs, signed informed consent was obtained from the parent or guardian of each participating child.

### 2.2. Study Design and Specimen Collection

This was a retrospective study conducted between June and October 2021 at the Fifth Affiliated Hospital of Southern Medical University, the only Class A tertiary comprehensive hospital in Conghua District, Guangzhou, China. The study population was composed of pediatric patients (aged under 5 years) who were clinically suspected to have gastrointestinal disease (or non-typhoidal *Salmonella* infection). Anal swabs were collected for all participating children. Specimens were transported within one hour of collection to the microbiology laboratory for processing, culture, identification, and antimicrobial susceptibility testing.

### 2.3. Questionnaire

A structured questionnaire including socio-demographic characteristics (gender, age, and residence), common clinical presentations (diarrhea, abdominal pain, nausea, emesis, fever, cough), and factors that may increase the risk of *Salmonella* transmission (e.g., infant and child feeding, type of powdered milk used, preservation of complementary food, personal hygiene practices, and animals keeping) was administered to the guardians of each child. Each questionnaire was linked to one anal swab and was used for our analysis in the present study.

### 2.4. Salmonella Strains, DNA Extraction, and WGS

The anal swabs from the participants were processed and analyzed using standard microbiological methods, and suspected colonies were further identified using the VITEK-Compact 2 automatic microbial identification system (bioMérieux, Marcy-l’Étoile, France). The strains were subjected to serotyping methods using commercial O and H antigens (Ningbo Tianrun biopharmaceutical Co., Ltd., Ningbo, China), based on the Kauffmann–White–Le Minor scheme. The genomic DNA of *Salmonella* isolates was extracted using the bacterial genomic DNA extraction kit (Tiangen, Beijing, China), according to the manufacturer’s recommended procedures [23]. Sequencing of the paired-end libraries (2 × 150 bp) was executed on a HiSeq 2500 instrument (Illumina, San Diego, CA, USA). The quality of the raw sequences was examined using FastQC, the read sequences were assembled using SPAdes 3.13.1, and all genomes were annotated by Prokka 1.14.5 [24,25,26]. The serotypes and STs of the NTS strains were analyzed using SISTR 1.1.1 and MLST 2.18.0, respectively [27,28]. AMR gene detection was conducted based on the Center for Genomic Epidemiology ResFinder 4.1 webtool, with the default thresholds of a 90% nucleotide sequence identity and a 60% minimum length [29]. A phylogenetic tree was constructed using RAXML v8.1.23, with the model GTRGAMMA [30]. The reliability of the tree was assessed by bootstrap analysis with 100 replicates. The complete genome of NTS obtained in the present study was deposited in the GenBank database under the following accession numbers: JAVGDA000000000–JAVGIY000000000.

### 2.5. Antimicrobial Susceptibility Testing

The obtained bacterial isolates were evaluated for susceptibility to the five antimicrobial agents using the disk diffusion method (Kirby–Bauer), as recommended by the Clinical and Laboratory Standards Institute (CLSI). The following antimicrobial agents and concentration were used: azithromycin (AZM, 15 mg), ampicillin (AMP, 10 mg), ciprofloxacin (CIP, 5 mg), chloramphenicol (CHL, 30 g), and tetracycline (TCY, 30 μg). The minimum inhibitory concentrations (MICs) were determined by broth microdilution for 14 antibiotics: amoxicillin-clavulanate (AMC), ertapenem (ETP), trimethoprim-sulfamethoxazole (SXT), piperacillin/tazobactam (TZP), tigecycline (TGC), cefepime (FEP), cefuroxime (CXM), cefuroxime axetil (CXA), ceftriaxone (CRO), ceftazidime (CAZ), cefoxitin (FOX), imipenem (IPM), levofloxacin (LVX), and cefoperazone/sulbactam (CSL). The reading and interpretation of the results of the antibiotic susceptibility testing, labeled as sensitive, intermediate, and resistant, were conducted in reference to the CLSI 2021 criteria. In addition, the interpretive criteria for cefoperazone-sulbactam disks were the same as those for cefoperazone alone; the following MIC breakpoints should be used for the combination (2:1 ratio): ≥64/32 μg/mL, resistant; 32/16 μg/mL, moderately susceptible; and ≤16/8 μg/mL, susceptible [31] The NTS serotypes recognized by SISTR were classified as MDR and non-MDR serotypes.

### 2.6. Quality Control

Standard operating procedures were strictly followed during the course of anal swab collection, transportation, preservation, processing, and culturing. *Escherichia coli* ATCC 25922 was used as a quality control for antibiotic susceptibility testing in the present study.

### 2.7. Statistical Analysis

In the present study, Pearson Chi-square (χ^2^) and Fisher’s exact tests, based on the Statistical Package for the Social Sciences (SPSS) 19.0, were used to determine the relationships between the prevalence of NTS and the variables listed in Tables S1 and S2. All results were interpreted using odds ratios, 95% confidence intervals, and significance level (*p* value < 0.05). In addition, a *t*-test was used to compare the number of resistance genes between ST34 and ST19, and *p* < 0.05 was considered significant.

## 3. Results

### 3.1. Prevalence of NTS

The anal swabs collected from 164 (35.27%) out of 465 pediatric patients were positive for NTS in the present study. All 465 questionnaires were used for assessment of possible risk factors for NTS infection, as listed in Table S1. The prevalence of NTS was statistically different in four categories of possible risk factors: artificial feeding (artificial milk) (41.14%, 123/299) versus breastfeeding (10.81%, 4/37) (*p* = 0.0003), mixed feeding (both artificial feeding and breastfeeding) (37.33%, 28/75) versus breastfeeding (10.81%, 4/37) (*p* = 0.003), holding food room temperature (58.11%, 43/74) versus cooking for immediate consumption (36.08%, 92/255) (*p* = 0.001), and not washing hands (48.08%, 25/52) versus washing hands after using toilets (33.66%, 139/413) (*p* < 0.05).

In the present study, approximately 58% (n = 268) of patients presented with at least one of the six clinical symptoms (diarrhea, abdominal pain, nausea, emesis, fever, and cough) listed in Table S2. The most common presenting symptom was diarrhea (41.08%, 191/465), followed by fever (31.61%, 147/465), and cough (14.62%, 68/465). Of the patients with NTS infection, 71 (43.29%) presented with fever, and fever is a common symptom that may be caused by NTS infection (*p* < 0.001).

### 3.2. Serotyping, MLST, and Phylogenetic Analysis

The serotyping of NTS revealed that *S. 1,4,[5],12:i:-* (65.24%, 107/164) was the predominant serotype, followed by *S. Typhimurium* (15.24%, 25/164), *S. Stanley* (5.49%, 9/164), *S. Derby* (4.88%, 8/164), *S. Enteritidis* (3.05%, 5/164), *S. Rissen* (2.44%, 4/164), *S. London* (1.22%, 2/164), *S. Bareilly* (0.61%, 1/164), *S. Infantis* (0.61%, 1/164), *S. Corvallis* (0.61%, 1/164), and *S. Goldcoast* (0.61%, 1/164) (Table 1).

To estimate the genetic correlations, MLST was performed, based on seven housekeeping genes (aroC, dnaN, hemD, hisD, purE, sucA and thrA), and 164 strains were further classified into 12 individual STs (Table 1). Among them, ST34 (n = 103) and ST19 (n = 28) were the most common STs, comprising 62.80% and 17.07% of the whole strains. In addition, 9 strains with ST29 (5.49%), 8 strains with ST40 (4.88%), 5 strains with ST11 (3.05%), 4 strains with ST469 (2.44%), 2 strains with ST155 (1.22%), and 1 strain each with ST32 (0.61%), ST203 (0.61%), ST358 (0.61%), ST9826 (0.61%) and a novel ST (0.61%) were also identified. By assessing the association between MLST and the serotyping of 164 NTS strains, most serotypes correspond to one MLST type, with the exception of *S*. *1,4,[5],12:i:-* and *S*. *Typhimurium*. ST34 and ST19 are the most abundant ST of *S*. *1,4,[5],12:i:-* (96.26%, 103/107) and *S*. *Typhimurium* (96%, 24/25), respectively. In addition, Figure S1 shows the relationship between different STs and possible risk factors for NTS infection, as well as the clinical symptoms. Although the prevalence of STs differed within each of the group categories, no relationship was found between the STs and the risk factors studied, nor with the clinical symptoms.

**Figure 1:**
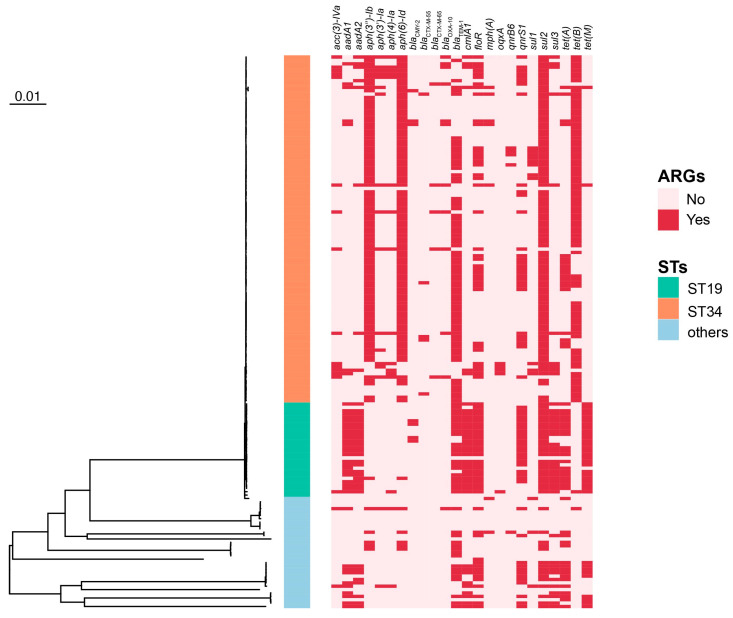
The phylogenetic tree of 164 NTS strains. Red boxes indicate the presence of an antibiotic resistance gene. ARGs: antibiotic resistance genes; STs: subtypes.

To better assess the genetic relationship of all the serotypes of NTS obtained in this study, a phylogenetic tree was constructed. The results showed the division of 132 isolates (*S. 1,4,[5],12:i:-* and *S. Typhimurium*) into two clades: one clade consists of 103 isolates, all of which were *S*. *1,4,[5],12:i:-* ST34, and the other one includes the remaining 29 isolates, consisting of 24 *S*. *Typhimurium* ST19, one *S*. *Typhimurium* novel ST, and 4 *S*. *1,4,[5],12:i:-* ST19 (Figure 1). The novel ST differs from ST19 in the locus of thrA (2 → 797).

### 3.3. Antibiotic Susceptibility Pattern and Resistance Genes

**Figure 2:**
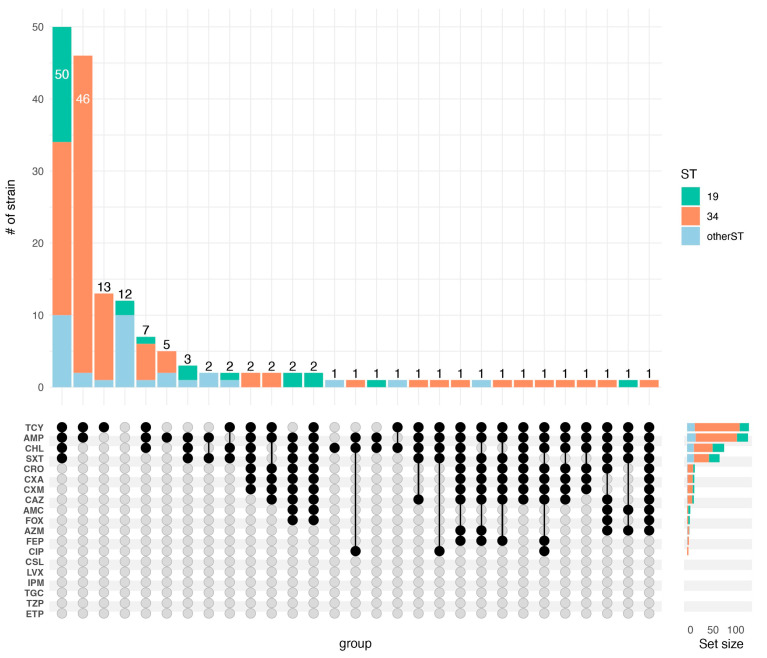
Distribution of NTS strains resistant to antibiotics, as well as the resistant spectrum. The top section indicates the number of strains corresponding to each resistance spectrum. For example, the first vertical bar indicates that there are 50 strains resistant to TCY-AMP-CHL-SXT, which are identified as ST19, ST34, and other subtypes.

According to the results of the antimicrobial susceptibility test for 164 strains of NTS, the most common phenotypic resistances were to AMP (82.3%), TCY (83.5%), CHL (50.0%), and SXT (43.9%) (Table 2). Six antibiotics of the cephalosporin class were used for testing, and 2.4%, 9.8%, 9.8%, 10.4%, 9.1%, and 3.7% strains were resistant to FEP, CXM, CXA, CRO, CAZ, and FOX, respectively. No strains were found to be resistant to ETP, TZP, TGC, IPM, LVX, or CSL (Table 2). For most of the antibiotics tested in this study, ST19 was associated with a higher resistance rate than ST34 (Table S3). Of the 164 NTS isolates, the susceptibility results showed that all of the isolates were resistant to at least one antimicrobial, and 50.61% (83/164) of the isolates were MDR strains. TCY-AMP-CHL-SXT (30.49%, 50/164) was the predominant resistance spectrum (Figure 2).

The WGS-based genotypic analysis revealed 69 different resistance genes (Excels S1 and S2). The sulfanilamide resistance *sul2* gene, the *beta-lactam*_TEM-1_ gene, and the aminoglycoside resistance *aph(6)-Id*, *aph(3”)-Ib* genes were frequently detected in the tested isolates (84.15%, 67.68%, 61.59%, and 60.98% respectively). Genes which are responsible for resistance to tetracycline (*tetB* and *tetA*) (54.27% and 33.54%), chloramphenicol (*floR*) (48.78%), and fluoroquinolone (*qnrS1*) (39.02%) were also widely distributed (Figure 1).

Among 17 ceftriaxone-resistant strains, the predominant serotype was *S. 1,4,[5],12:i:-* (70.59%, 12/17) (Excel S3). All of these ceftriaxone-resistant strains were MDR, and 11 kinds of β-lactamases genes were detected. Among them, *bla*_TEM-1_ (n = 7), *bla*_CMY-2_ (n = 6), and *bla*_OXA-10_ (n = 6) were the main β-lactamase genes.

## 4. Discussion

In the present study, the NTS infection rate of children under 5 years old was 35.27%, which was slightly lower than that obtained by the survey conducted in Hong Kong (41.91%) [32], but much higher than the survey conducted at Guangzhou Maternal and Child Medical Center (11.23%) [33]. This could be because the participants in the study by Woh et al. [32] were children under 5 years of age hospitalized with gastroenteritis. However, in the study by Gao et al. [33], the participants were children of all ages, and samples were collected throughout the year. It is widely known that *Salmonella* is one of the leading causes of diarrhea in children under 5 years of age, and it most commonly occurs between summer and autumn [34,35]. The high frequency of infections in children could be attributed to their immature immune systems, weak gastrointestinal systems, poor hygiene practices, and child-specific behaviors (for example, crawling on the floor) [13,36]. Furthermore, high temperatures and heavy rainfall are positively associated with NTS infection [37,38]. These meteorological variations can enhance bacterial replication and transmission to surface water and food crops, which serve as potential sources of infection [39].

The results of this study are consistent with those obtained in some previous studies showing that breastfeeding is associated with a decreased risk of NTS infection [40,41,42]. The protective effect of breastfeeding may be explained by the following two points: phagocytic cells in colostrum and breast milk are active against *Salmonella*; thus, breast milk can provide host defense for infants. Another possible explanation is that dietary exposures are limited in exclusively breastfed infants, avoiding the risks inherent in infant contact with powdered formula [40]. Powdered infant formula is not a sterile product, and it may be intrinsically contaminated with *Salmonella*. There are more than 10 documented outbreaks of *Salmonella* infection in infants that have been linked to ingestion of powdered infant formula [41]. In addition, improperly stored food is also a risk factor for *Salmonella* infection [43]. In Europe, 25% of the food-borne outbreaks of *Salmonella* are due to inappropriate storage and preparation of food [43].

The most frequently identified serotypes in the present study were *S*. *1,4,[5],12:i:-* (65.24% of all strains) and *S*. *Typhimurium* (15.24% of all strains). However, the detection rate of *S*. *Enteritidis* was only 3.05%. Epidemiological data have revealed that the distribution of NTS serotypes in humans is different among geographic areas, and the complex group of *S*. *Typhimurium* and 4,5,12:i:- ranked first in the endemic serotypes of *Salmonella* in South China [22]. *S*. *1,4,[5],12:i:-* is a monophasic variant of *S*. *Typhimurium* that lacks the fljB-encoded second-phase H antigen. The proportion of *S*. *1,4,[5],12:i:-* has shown an increasing trend since 2011, while exceeding the proportion of *S*. *Typhimurium* in 2016 [22]. Another possible explanation for the high detection of *S*. *1,4,[5],12:i:-* in this study is that the subjects were children aged under 5 years. In the period of 2011–2021, of the more than 4100 strains of *S*. *1,4,[5],12:i:-* isolated from humans in China, 74% were obtained from infants and children under 3 years old [44].

Foodborne diseases caused by *S*. *Typhimurium* and *S*. *1,4,[5],12:i:-* are primarily associated with pigs and pork products [22]. In Guangdong Province, it is customary for parents to feed congee mixed with cooked minced pork and steamed egg to infants during the weaning stage [45]. Moreover, Cantonese sausage is a type of sausage that many locals eat often, and the main ingredient is pork. Although no information is available on *Salmonella* infection in congee and Cantonese sausage, some studies have reported the occurrence of *S*. *Typhimurium* and *S*. *1,4,[5],12:i:-* in retail meats and other types of sausages [46,47]. Therefore, *S*. *Typhimurium* and *S*. *1,4,[5],12:i:-* may be transmitted through pork meat and pork meat products, a fact requiring further research in the future. Several other serotypes (such as *S*. *Stanley*, *S*. *Derby*, and *S*. *Infantis*), common in many countries, were also found in this study, which may be related to the frequent global travel, communication, and trading that occur in Guangdong [48].

The 164 isolates were assigned to 12 STs using MLST, of which ST34 (62.80%) and ST19 (17.07%) were the most common STs found in this study. Despite the fact that ST19 is the most prevalent ST from the MLST database, ST34 has replaced ST19 as the dominant ST in China in recent years [22,49]. Pediatric and adult NTS infection were mainly caused by ST34 from pigs and ST19 from chickens, respectively [22]. It was reported that contamination by *Salmonella* in livestock and poultry products in Guangdong Province is quite serious, and these contaminated food are considered as the primary source of human infection [49,50]. Therefore, we should pay more attention to the monitoring of *Salmonella* in the animal-origin food supply.

With regard to clinical manifestations, we found that fever and diarrhea were the prominent symptoms. These results are in agreement with those of previous studies in Guangdong and Fujian Provinces in China [45,51]. Therefore, the importance of *Salmonella* detection in the diagnosis and treatment of children should be emphasized to avoid misdiagnosis.

**Figure 3:**
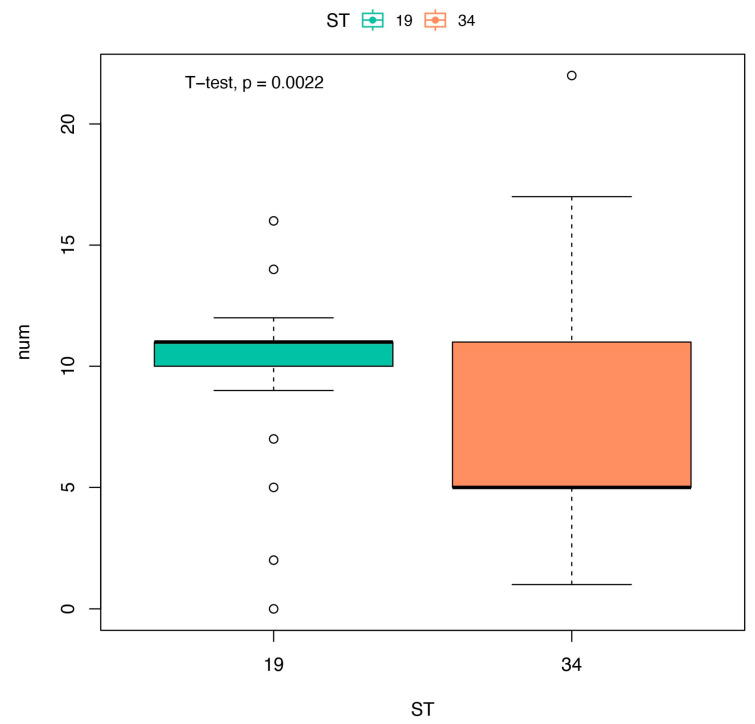
The number of resistance genes carried by different subtypes. T-values of the *t*-test for independent samples, with subtypes as the group variables and the number of resistance genes as dependent variable. t = 3.2109; *p* = 0.0022.

High antimicrobial resistance rates of conventional first-line agents for treatment of severe salmonellosis were observed in this study, and 82.3%, 83.5%, 50.0%, and 43.9% of the isolates displayed resistance to AMP, TCY, CHL, and SXT, respectively. Due to the development of resistance, these first-line antimicrobials have become less effective and should be used less frequently [52]. Extended-spectrum cephalosporins and fluoroquinolones are currently the two crucial antimicrobials for salmonellosis treatment [53]. However, due to potential adverse effects, fluoroquinolones and aminoglycosides are not typically used in children [13]. Cephalosporins, especially the third-generation cephalosporins, are usually the drugs of choice for salmonellosis treatment in children [54]. The susceptibility tests showed that the rate of resistance to CRO was 10.4%, which was lower than that found in previous domestic studies conducted in children (24.5%, 37.4%, and 40.1% in Fujian, Zhejiang, and Guangdong Province, respectively) [33,38,45]. It is known that CTX-M is the most common extended-spectrum beta-lactamase gene worldwide that preferentially hydrolyzes CRO or cefotaxime, resulting in *Salmonella* resistance to broad-spectrum cephalosporin [55,56]. Therefore, the low rate of resistance to third-generation cephalosporins (such as CRO) may be related to the low detection rate of *bla*_CTX-M_ in this study. In fact, the drug resistance mechanism of *Salmonella* is very complex, requiring further explored in our future studies. In addition, the high level of antibiotic resistance observed in ST19 may be related to the higher number of resistance genes in ST19 than in ST34 (*p* < 0.01) (Figure 3). Therefore, it is necessary to further study the association between STs and drug resistance to provides useful information for epidemiological research and to develop a public health strategy.

In general, the current resistance rate of third-generation cephalosporins is still low, but the proportion of MDR strains is high (50.61%); and 44.86% (48/107) of *S*. *1,4,[5],12:i:-* isolates were MDR. According to previous studies, there are two primary MDR clones (European clone and Spanish clone) of *S*. *1,4,[5],12:i:-* [57]. Our results are basically consistent with the characteristics of European clones, which are resistant to ASSuT. Meanwhile, the resistance gene results showed that most isolates harbored the *sul2*, *bla*_TEM-1_, and *tetB* genes, which are typically associated with the European clone [57]. Therefore, the MDR clone of the *S*. *1,4,[5],12:i:-* isolates obtained in this study might be related to the European clone. Overall, the emergence of MDR *Salmonella* isolates, particularly those resistant to third-generation cephalosporins, may lead to failure in the treatment of *Salmonella* infections in children. Therefore, the treatment of salmonellosis should be combined with drug susceptibility results to avoid the occurrence of MDR strains resulting from the excessive or inappropriate use of clinical antibiotics.

The virulence factors carried by the strains were not analyzed in this study. The pathogenicity of *Salmonella* results from the interaction of numerous virulence factors. Therefore, further analysis of the virulence phenotype and genomic characteristics of *Salmonella* should be conducted in future studies to understand its virulence mechanism and reduce its pathogenicity. Although the data comes from a single hospital, the Fifth Affiliated Hospital of Southern Medical University is the largest hospital in northern Guangzhou. Therefore, the data can reflect the real epidemiology of Conghua District, Guangzhou.

## 5. Conclusions

The present study demonstrated the prevalence, genetic characteristics, and antimicrobial resistance of NTS in children aged < 5 years in a tertiary hospital in Guangzhou, China. A total of 35.27% of the participants were found to be positive for NTS, with 11 serotypes being identified. Breastfeeding, eating fresh food, and establishing good hygiene habits are associated with a decreased risk of NTS infection. *S*. *1,4,[5],12:i:-* and ST34 were detected as the most common serotype and subtype, respectively. High antimicrobial resistance rates were observed for the conventional first-line antimicrobials (AMP, TCY, CHL, and SXT), while the resistance rate of cephalosporins (FEP, CXM, CXA, CRO, CAZ, and FOX) was relatively low. The high burden of MDR strains highlights the necessity of surveillance for the MDR clone to prevent its further dissemination in the investigated areas. In addition, antimicrobial resistance may be associated with different STs; therefore, a more comprehensive exploration of resistance mechanisms should be performed in future studies.

## Figures and Tables

**Table 1:** Subtypes corresponding to each NTS serotype in 164 strains.

Serogroup (n)	Serotype (n)	ST (n)
B (149)	*S. 1,4,[5],12:i:-* (107)	ST34 (103), ST19 (4)
	*S. Typhimurium* (25)	ST19 (24), novel ST (1)
	*S. Stanley* (9)	ST29 (9)
	*S. Derby* (8)	ST40 (8)
C1 (6)	*S. Rissen* (4)	ST469 (4)
	*S. Bareilly* (1)	ST203 (1)
	*S. Infantis* (1)	ST32 (1)
C2–C3 (2)	*S. Corvallis* (1)	ST9826 (1)
	*S. Goldcoast* (1)	ST358 (1)
D (5)	*S. Enteritidis* (5)	ST11 (5)
E1 (2)	*S. London* (2)	ST155 (2)

**Table 2:** Antimicrobial resistance pattern of NTS strains identified in this study (N = 164).

Antimicrobial	Resistant %	Intermediate %	Susceptible %
AMC	4.3	8.5	87.2
AZM	3	25	72
AMP	82.3	1.8	15.9
ETP	0	0	100
SXT	43.9	0	56.1
CIP	4.3	55.5	40.2
CHL	50	0	50
TZP	0	0.6	99.4
TCY	83.5	1.2	15.2
TGC	0	6.7	93.3
FEP	2.4	1.8	95.7
CXM	9.8	1.8	88.4
CXA	9.8	4.3	86
CRO	10.4	0	89.6
CAZ	9.1	0	90.9
FOX	3.7	0	96.3
IPM	0	0	100
LVX	0	3	97
CSL	0	1.2	98.8

AMC: amoxicillin-clavulanate; AZM: azithromycin; AMP: ampicillin; ETP: ertapenem; SXT: trimethoprim-sulfamethoxazole; CIP: ciprofloxacin; CHL: chloramphenicol; TZP: piperacillin/tazobactam; TCY: tetracycline; TGC: tigecycline; FEP: cefepime; CXM: cefuroxime; CXA: cefuroxime axetil; CRO: ceftriaxone; CAZ: ceftazidime; FOX: cefoxitin; IPM: imipenem; LVX: levofloxacin; CSL: cefoperazone/sulbactam.

## Data Availability

The data are contained within the article or Supplementary Materials.

## Supplementary Materials

The following supporting information can be downloaded at: https://www.mdpi.com/article/10.3390/microorganisms11102433/s1, Figure S1: Relationship between different subtypes and possible risk factors for NTS infection, as well as clinical symptoms; Table S1: Assessment of possible risk factors for NTS infection (N = 465); Table S2: Relationship between NTS infection and clinical symptoms; Table S3: Antimicrobial resistance pattern of ST34 (N = 103) and ST19 (N = 28) strains, respectively; Excel S1: Distribution of resistance genes among different STs; Excel S2: Antibiotic resistance genes in each strain; Excel S3: Serotypes, subtypes, and β-Lactamase resistance genes of ceftriaxone-resistant strains.

## References

[1] GBD 2016 Diarrhoeal Disease Collaborators (2018). Estimates of the global, regional, and national morbidity, mortality, and aetiologies of diarrhoea in 195 countries: A systematic analysis for the Global Burden of Disease Study 2016. Lancet. Infect. Dis..

[2] WHO. https://www.who.int/news-room/fact-sheets/detail/salmonella-(non-typhoidal).

[3] Wu L.J., Luo Y., Shi G.L., Li Z.Y. (2021). Prevalence, clinical characteristics and changes of antibiotic resistance in children with nontyphoidal *Salmonella* infections from 2009–2018 in Chongqing, China. Infect. Drug. Resist..

[4] Mukherjee N., Nolan V.G., Dunn J.R., Banerjee P. (2019). Sources of human infection by *Salmonella* enterica serotype Javiana: A systematic review. PLoS ONE.

[5] Jackson B.R., Griffin P.M., Cole D., Walsh K.A., Chai S.J. (2013). Outbreak-associated *Salmonella* enterica serotypes and food commodities, United States, 1998–2008. Emerg. Infect. Dis..

[6] Müller L., Kjelsø C., Frank C., Jensen T., Torpdahl M., Søborg B., Dorleans F., Rabsch W., Prager R., Gossner C.M. (2016). Outbreak of *Salmonella* Strathcona caused by datterino tomatoes, Denmark, 2011. Epidemiol. Infect..

[7] Marshall K.E.H., Tewell M., Tecle S., Leeper M., Sinatra J., Kissler B., Fung A., Brown K., Wagner D., Trees E. (2018). Protracted outbreak of *Salmonella* newport infections linked to ground beef: Possible role of dairy cows—21 States, 2016–2017. MMWR Morb. Mortal. Wkly. Rep..

[8] Laughlin M., Bottichio L., Weiss J., Higa J., McDonald E., Sowadsky R., Fejes D., Saupe A., Provo G., Seelman S. (2019). Multistate outbreak of *Salmonella* Poona infections associated with imported cucumbers, 2015–2016. Epidemiol. Infect..

[9] Sloan-Gardner T.S., Waters N., Marmor A., Mude W. (2019). Free range eggs does not mean safe eggs: An outbreak of *Salmonella* Typhimurium linked to free range eggs. Commun. Dis. Intell..

[10] Laidlow T.A., Stafford R., Jennison A.V., Bell R., Graham R., Graham T., Musgrave N., Myerson M., Kung N., Crook A. (2022). A multi-jurisdictional outbreak of *Salmonella* Typhimurium infections linked to backyard poultry-Australia, 2020. Zoonoses. Public. Health..

[11] Butler A.J., Thomas M.K., Pintar K.D.M. (2015). Expert elicitation as a means to attribute 28 enteric pathogens to foodborne, waterborne, animal contact, and person-to-person transmission routes in Canada. Foodborne. Pathog. Dis..

[12] Christidis T., Hurst M., Rudnick W., Pintar K.D.M., Pollari F. (2020). A comparative exposure assessment of foodborne, animal contact and waterborne transmission routes of *Salmonella* in Canada. Food. Control..

[13] Xu L., He Q., Tang Y., Wen W., Chen L., Li Y., Yi C., Fu B. (2022). Multi-locus sequence and drug resistance analysis of Salmonella infection in children with diarrhea in Guangdong to identify the dominant ST and cause of antibiotic-resistance. Exp. Ther. Med..

[14] Centers for Disease Control and Prevention (2018). National Salmonella Surveillance Annual Report, 2016.

[15] European Food Safety Authority and European Centre for Disease Prevention and Control (EFSA and ECDC) (2018). The European Union summary report on trends and sources of zoonoses, zoonotic agents and food-borne outbreaks in 2017. EFSA J..

[16] Grattarola C., Gallina S., Giorda F., Pautasso A., Ballardini M., Iulini B., Varello K., Goria M., Peletto S., Masoero L. (2019). First report of *Salmonella 1,4,[5],12:i:*- in free-ranging striped dolphins (*Stenella coeruleoalba*), Italy. Sci. Rep..

[17] Zeng X.Y., Lv S.L., Qu C., Lan L., Tan D.M., Li X.G., Bai L. (2021). Serotypes, antibiotic resistance, and molecular characteriza-tion of non-typhoidal *Salmonella* isolated from diarrheic patients in Guangxi Zhuang Autonomous Region, China, 2014–2017. Food Control..

[18] Baker S., Thomson N., Weill F.X., Holt K.E. (2018). Genomic insights into the emergence and spread of antimicrobial-resistant bacterial pathogens. Science.

[19] Zhang H., Xiang Y., Huang Y., Liang B., Xu X., Xie J., Du X., Yang C., Liu H., Liu H. (2022). Genetic characterization of mcr-1-positive multidrug-resistant *Salmonella* enterica serotype Typhimurium isolated from intestinal infection in children and pork offal in China. Front. Microbiol..

[20] Wang X., Biswas S., Paudyal N., Pan H., Li X., Fang W., Yue M. (2019). Antibiotic resistance in *Salmonella* Typhimurium isolates recovered from the food chain through national antimicrobial resistance monitoring system between 1996 and 2016. Front. Microbiol..

[21] Li Y., Zhang Y., Chen M., Hu J., Zhang H., Xiang Y., Yang H., Qiu S., Song H. (2021). Plasmid-borne colistin resistance gene mcr-1 in a multidrug resistant *Salmonella* enterica serovar Typhimurium isolate from an infant with acute diarrhea in China. Int. J. Infect. Dis..

[22] Wang Y., Liu Y., Lyu N., Li Z., Ma S., Cao D., Pan Y., Hu Y., Huang H., Gao G.F. (2022). The temporal dynamics of antimicrobial-resistant *Salmonella* enterica and predominant serovars in China. Natl. Sci. Rev..

[23] Zeng S., Zhuo Z., Huang Y., Luo J., Feng Y., Gong B., Huang X., Wu A., Zhuo C., Li X. (2022). Prevalence of chromosomally located blaCTX-M-55 in *Salmonella* Typhimurium ST34 isolates recovered from a tertiary hospital in Guangzhou, China. Microbiol. Spectr..

[24] Bioinformatics B. FastQC a Quality Control Tool for High Throughput Sequencedata. http://www.bioinformatics.babraham.ac.uk/projects/fastqc/.

[25] Bankevich A., Nurk S., Antipov D., Gurevich A.A., Dvorkin M., Kulikov A.S., Lesin V.M., Nikolenko S.I., Pham S., Prjibelski A.D. (2012). SPAdes: A new genome assembly algorithm and its applications to single-cell sequencing. J. Comput. Biol..

[26] Seemann T. (2014). Prokka: Rapid prokaryotic genome annotation. Bioinformatics.

[27] Larsen M.V., Cosentino S., Rasmussen S., Friis C., Hasman H., Marvig R.L., Jelsbak L., Sicheritz-Pontén T., Ussery D.W., Aarestrup F.M. (2012). Multilocus sequence typing of total-genome-sequenced bacteria. J. Clin. Microbiol..

[28] Yoshida C.E., Kruczkiewicz P., Laing C.R., Lingohr E.J., Gannon V.P., Nash J.H., Taboada E.N. (2016). The *Salmonella* in Silico Typing Resource (SISTR): An open web-accessible tool for rapidly typing and subtyping draft *Salmonella* genome assemblies. PLoS ONE.

[29] Thomsen M.C., Ahrenfeldt J., Cisneros J.L., Jurtz V., Larsen M.V., Hasman H., Aarestrup F.M., Lund O. (2016). A bacterial analysis platform: An integrated system for analysing bacterial whole genome sequencing data for clinical diagnostics and surveillance. PLoS ONE.

[30] Stamatakis A. (2014). RAxML version 8: A tool for phylogenetic analysis and post-analysis of large phylogenies. Bioinformatics.

[31] Jones R.N., Barry A.L., Packer R.R., Gregory W.W., Thornsberry C. (1987). In vitro antimicrobial spectrum, occurrence of synergy, and recommendations for dilution susceptibility testing concentrations of the cefoperazone-sulbactam combination. J. Clin. Microbiol..

[32] Woh P.Y., Yeung M.P.S., Goggins W.B., Lo N., Wong K.T., Chow V., Chau K.Y., Fung K., Chen Z., Ip M. (2021). Genomic epidemiology of multidrug-resistant nontyphoidal *Salmonella* in young children hospitalized for gastroenteritis. Microbiol. Spectr..

[33] Gao F., Huang Z., Xiong Z., Zheng H., Deng Q., Zhong H., Zhu S., Long Y., Wang J. (2023). Prevalence, serotype, and antimicrobial resistance profiles of children infected with *Salmonella* in Guangzhou, southern China, 2016–2021. Front. Pediatr..

[34] Deng X., Ran L., Wu S., Ke B., He D., Yang X., Zhang Y., Ke C., Klena J.D., Yan M. (2012). Laboratory-based surveillance of non-typhoidal *Salmonella* infections in Guangdong Province, China. Foodborne Pathog. Dis..

[35] Chen C., Wang L.P., Yu J.X., Chen X., Wang R.N., Yang X.Z., Zheng S.F., Yu F., Zhang Z.K., Liu S.J. (2019). Prevalence of enteropathogens in outpatients with acute diarrhea from urban and rural areas, southeast China, 2010–2014. Am. J. Trop. Med. Hyg..

[36] Shen H., Chen H., Ou Y., Huang T., Chen S., Zhou L., Zhang J., Hu Q., Zhou Y., Ma W. (2020). Prevalence, serotypes, and antimicrobial resistance of *Salmonella* isolates from patients with diarrhea in Shenzhen, China. BMC Microbiol..

[37] Wang P., Goggins W.B., Chan E.Y.Y. (2018). Associations of *Salmonella* hospitalizations with ambient temperature, humidity and rainfall in Hong Kong. Environ. Int..

[38] Ke Y., Lu W., Liu W., Zhu P., Chen Q., Zhu Z. (2020). Non-typhoidal *Salmonella* infections among children in a tertiary hospital in Ningbo, Zhejiang, China, 2012–2019. PLoS Negl. Trop. Dis..

[39] Jiang C., Shaw K.S., Upperman C.R., Blythe D., Mitchell C., Murtugudde R., Sapkota A.R., Sapkota A. (2015). Climate change, extreme events and increased risk of salmonellosis in Maryland, USA: Evidence for coastal vulnerability. Environ. Int..

[40] Rowe S.Y., Rocourt J.R., Shiferaw B., Kassenborg H.D., Segler S.D., Marcus R., Daily P.J., Hardnett F.P., Slutsker L., Emerging Infections Program FoodNet Working Group (2004). Breast-feeding decreases the risk of sporadic salmonellosis among infants in foodnet sites. Clin. Infect. Dis..

[41] Jones T.F., Ingram L.A., Fullerton K.E., Marcus R., Anderson B.J., McCarthy P.V., Vugia D., Shiferaw B., Haubert N., Wedel S. (2006). A case-control study of the epidemiology of sporadic *Salmonella* infection in infants. Pediatrics.

[42] Ehlayel M.S., Bener A., Abdulrahman H.M. (2009). Protective effect of breastfeeding on diarrhea among children in a rapidly growing newly developed society. Turk. J. Pediatr..

[43] Kovats R.S., Edwards S.J., Hajat S., Armstrong B.G., Ebi K.L., Menne B. (2004). The effect of temperature on food poisoning: A time-series analysis of salmonellosis in ten European countries. Epidemiol. Infect..

[44] Qin X., Yang M., Cai H., Liu Y., Gorris L., Aslam M.Z., Jia K., Sun T., Wang X., Dong Q. (2022). Antibiotic resistance of *Salmonella* Typhimurium monophasic variant 1,4,[5],12:i:-in China: A systematic review and meta-analysis. Antibiotics.

[45] Chen H., Qiu H., Zhong H., Cheng F., Wu Z., Shi T. (2023). Non-typhoidal *Salmonella* infections among children in Fuzhou, Fujian, China: A 10-year retrospective review from 2012 to 2021. Infect. Drug. Resist..

[46] Helmuth I.G., Espenhain L., Ethelberg S., Jensen T., Kjeldgaard J., Litrup E., Schjørring S., Müller L. (2019). An outbreak of monophasic *Salmonella* Typhimurium associated with raw pork sausage and other pork products, Denmark 2018–19. Epidemiol. Infect..

[47] Li C., Gu X., Zhang L., Liu Y., Li Y., Zou M., Liu B. (2022). The occurrence and genomic characteristics of mcr-1-harboring *Salmonella* from retail meats and eggs in Qingdao, China. Foods.

[48] Ke B., Sun J., He D., Li X., Liang Z., Ke C.W. (2014). Serovar distribution, antimicrobial resistance profiles, and PFGE typing of *Salmonella* enterica strains isolated from 2007–2012 in Guangdong, China. BMC Infect. Dis..

[49] Sun J., Ke B., Huang Y., He D., Li X., Liang Z., Ke C. (2014). The molecular epidemiological characteristics and genetic diversity of *Salmonella* Typhimurium in Guangdong, China, 2007–2011. PLoS ONE.

[50] Zhang L., Fu Y., Xiong Z., Ma Y., Wei Y., Qu X., Zhang H., Zhang J., Liao M. (2018). Highly prevalent multidrug-resistant *Salmonella* from chicken and pork meat at retail markets in Guangdong, China. Front. Microbiol..

[51] Liang Z., Ke B., Deng X., Liang J., Ran L., Lu L., He D., Huang Q., Ke C., Li Z. (2015). Serotypes, seasonal trends, and antibiotic resistance of non-typhoidal *Salmonella* from human patients in Guangdong Province, China, 2009–2012. BMC Infect. Dis..

[52] Gong B., Li H., Feng Y., Zeng S., Zhuo Z., Luo J., Chen X., Li X. (2022). Prevalence, Serotype distribution and antimicrobial resistance of non-typhoidal *Salmonella* in hospitalized patients in Conghua district of Guangzhou, China. Front. Cell. Infect. Microbiol..

[53] Long L., You L., Wang D., Wang M., Wang J., Bai G., Li J., Wei X., Li S. (2022). Highly prevalent MDR, frequently carrying virulence genes and antimicrobial resistance genes in *Salmonella* enterica serovar 4,[5],12:i:- isolates from Guizhou Province, China. PLoS ONE.

[54] Egorova A., Shelenkov A., Kuleshov K., Kulikova N., Chernyshkov A., Manzeniuk I., Mikhaylova Y., Akimkin V. (2023). Plasmid Composition, Antimicrobial resistance and virulence genes profiles of ciprofloxacin- and third-generation cephalosporin-resistant foodborne *Salmonella* enterica isolates from Russia. Microorganisms.

[55] He D., Chiou J., Zeng Z., Liu L., Chen X., Zeng L., Chan E.W., Liu J.H., Chen S. (2015). Residues distal to the active site contribute to enhanced catalytic activity of variant and hybrid β-Lactamases derived from CTX-M-14 and CTX-M-15. Antimicrob. Agents Chemother..

[56] Yue M., Liu D., Li X., Jin S., Hu X., Zhao X., Wu Y. (2022). Epidemiology, serotype and resistance of *Salmonella* isolates from a children’s hospital in Hangzhou, Zhejiang, China, 2006–2021. Infect. Drug. Resist..

[57] Sun H., Wan Y., Du P., Bai L. (2020). The epidemiology of monophasic *Salmonella* Typhimurium. Foodborne. Pathog. Dis..

