# Supplementary material for: Epidemiological, Genetic, and Phenotypic Characteristics of Non-Typhoidal Salmonella in Young Children, as Obtained from a Tertiary Hospital in Guangzhou, China
Source: Microorganisms. 2023 Sep 28;11(10):2433. doi: 10.3390/microorganisms11102433 (PMC10609151; doi:10.3390/microorganisms11102433)
Supplement: Supplementary file 1 [file microorganisms-11-02433-s001.zip › Supplementary Figure S1.pdf]

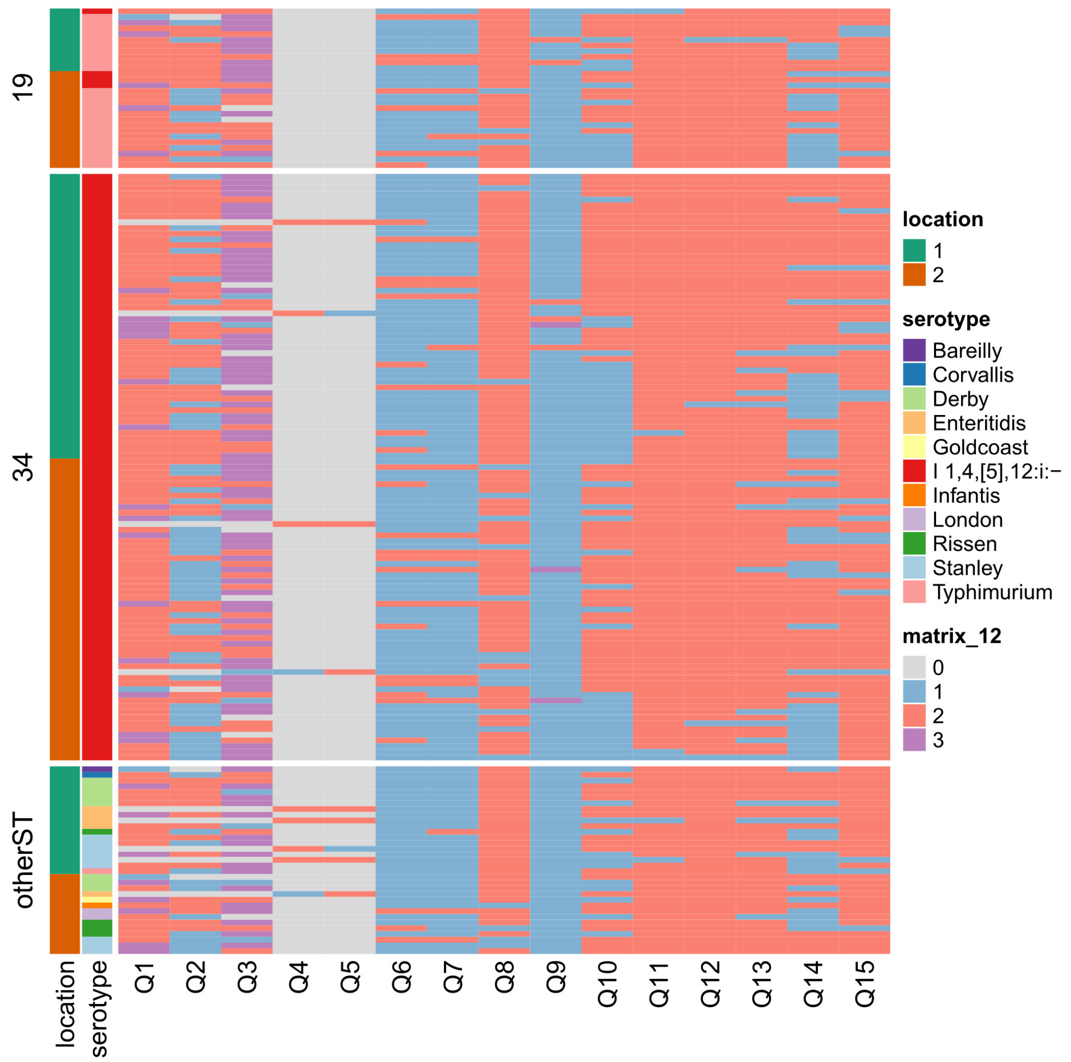

Figure S1. Relationship between different subtypes and possible risk factors for NTS infection as well as clinical symptoms. Q1: infant and child feeding; Q2: type of powdered milk; Q3: preservation of complementary food; Q4: eating unwashed vegetables and fruits; Q5: separate chopping boards for handling raw and cooked food; Q6: washing hands before meals; Q7: washing hands after using toilets; Q8: contact with animals frequently; Q9: nutritional status; Q10: diarrhea; Q11: abdominal pain; Q12: nausea; Q13: Emesis; Q14: fever; Q15: cough. For Q1: 1: breastfeeding, 2: artificial feeding, 3: both; For Q3: 1: refrigerated, 2: room temperature, 3: cook for immediate consumption; For other Questions: 0: not applicable, 1: yes, 2: no.
