# Supplementary material for: Epidemiological, Genetic, and Phenotypic Characteristics of Non-Typhoidal Salmonella in Young Children, as Obtained from a Tertiary Hospital in Guangzhou, China
Source: Microorganisms. 2023 Sep 28;11(10):2433. doi: 10.3390/microorganisms11102433 (PMC10609151; doi:10.3390/microorganisms11102433)
Supplement: Supplementary file 1 [file microorganisms-11-02433-s001.zip › Supplementary Tables S1-S3.pdf]

Table S1. Assessment of possible risk factors for NTS infection (N = 465).

**Variable**		**Examined no.**	**Positive no. (%)**	**OR (95% CI)[a]**	**χ^2/ p value**
Gender	Male	289	96 (33.22)	0.790 (0535, 1.167)	1.407/ 0.236
	Female	176	68 (38.64)		
Age (years)	Infants (0–1)	237	85 (35.86)	0.948 (0.648, 1.387)	0.075/ 0.784
	Children (2–5)	228	79 (34.65)		
Residence	Urban	224	80 (35.71)	1.038 (0.710, 1.519)	0.038/ 0.846
	Village	241	84 (34.85)		
Infant and child feeding	Breastfeeding	37	4 (10.81)	ref	
	Artificial feeding	299	123 (41.14)	5.776 (1.992, 16.691)	**12.880/ 0.0003[b]**
	Both	75	28 (37.33)	4.915 (1.575, 15.341)	**8.540/ 0.003[b]**
Type of powdered milk	Domestic	175	68 (38.86)	0.888 (0.587, 1.344)	0.314/ 0.575
	Imported	199	83 (41.71)		
Preservation of complementary food	Refrigerated	24	10 (41.67)	0.790 (0.337, 1.850)	0.295/0.587
	Room temperature	74	43 (58.11)	0.407 (0.240, 0.690)	**11.504/ 0.001[b]**
	Cook for immediate consumption	255	92 (36.08)	ref	
Eating unwashed vegetables and fruits	Yes	14	2 (14.29)	1.273 (0.231, 7.000)	0/ 1.000
	No	40	7 (17.50)		
Separate chopping boards for handling raw and cooked food	Yes	24	2 (8.33)	3.348 (0.626, 17.903)	1.215/ 0.270
	No	30	7 (23.33)		
Washing hands before meals	Yes	389	132 (33.93)	1.416 (0.858, 2.338)	1.860/ 0.173
	No	76	32 (42.11)		
Washing hands after using toilets	Yes	413	139 (33.66)	1.825 (1.021, 3.263)	**4.207/ 0.04[b]**
	No	52	25 (48.08)		

Contact with animals frequently	Yes	54	22 (40.74)	0.768 (0.430, 1.371)	0.801/ 0.371
	No	411	142 (34.55)		
Animal species[c]	Poultry (eg. chickens and ducks)	22	10 (45.45)	ref	
	Companion animals (eg. dogs and cats)	33	15 (45.45)	1.000 (0.338, 2.955)	0/ 1.000
	Others (eg. reptiles and birds)	5	0	1.833 (1.252, 2.685)	–/ 0.124
Nutritional status	Good	447	156 (34.90)	ref	
	Fair	11	5 (45.45)	1.554 (0.467, 5.175)	0.525/ 0.469
	Poor	7	3 (42.86)	0.715 (0.158, 3.234)	0.192/ 0.661

[a]*CI, confidence interval.*

[b]*Bold type for values indicates statistical significance.*

[c]*Six children (including three NTS-positive children) are frequently exposed to both poultry and companion animals.*

Table S2. Relationship between NTS infection and clinical symptoms.

**Variable**		**All participants**			
		**Examined no.**	**Positive no. (%)**	**OR (95% CI)[b]**	**χ^2/ p value**
Clinical symptoms[a]	Yes	268	95 (35.45)	1.019 (0.693, 1.497)	0.009/ 0.925
	No	197	69 (35.03)		
Diarrhea	Yes	191	68 (35.60)	1.025 (0.697, 1.509)	0.016/ 0.900
	No	274	96 (35.04)		
Abdominal pain	Yes	27	6 (22.22)	0.506 (0.200, 1.281)	2.137/ 0.144
	No	438	158 (36.07)		
Nausea	Yes	10	4 (40.00)	1.229 (0.342, 4.420)	0/ 1.000
	No	455	160 (35.16)		
Emesis	Yes	37	16 (43.24)	1.441 (0.730, 2.846)	1.120/ 0.290
	No	428	148 (34.58)		
Fever	Yes	147	71 (48.30)	2.260 (1.509, 3.385)	**15.987/ 0.00006**[c]
	No	318	93 (29.25)		
Cough	Yes	68	23 (33.82)	0.928 (0.539, 1.597)	0.073/ 0.787
	No	397	141 (35.52)		

[a]*Indicating people who had at least one of the six symptoms listed in the present study.*
[b]*CI, confidence interval.*
[c]*Bold type for values indicates statistical significance.*

Table S3. Antimicrobial resistance pattern of ST34 (N = 103) and ST19 (N = 28) strains, respectively.

Antimicrobial	ST34			ST19			Total[a]		
	R %	I %	S %	R %	I %	S %	R %	I %	S %
AMC	1.9	6.8	91.3	17.9	3.6	78.6	5.3	6.1	88.6
AZM	2.9	27.2	69.9	3.6	17.9	78.6	3.8	25	71.2
AMP	88.3	1	10.7	89.3	3.6	7.1	88.6	1.5	9.8
ETP	0	0	100	0	0	100	0	0	100
SXT	32	0	68	85.7	0	14.3	43.9	0	56.1
CIP	5.8	48.5	45.6	3.6	82.1	14.3	5.3	56.1	38.6
CHL	39.8	0	60.2	92.9	0	7.1	50.8	0	49.2
TZP	0	0	100	0	3.6	96.4	0	0.8	99.2
TCY	96.1	0	3.9	75	3.6	21.4	91.7	0.8	7.6
TGC	0	8.7	91.3	0	0	100	0	6.8	93.2
FEP	2.9	2.9	94.2	0	0	100	3	2.3	94.7
CXM	10.7	1	88.3	14.3	0	85.7	12.1	0.8	87.1
CXA	10.7	1.9	87.4	14.3	0	85.7	12.1	1.5	86.4
CRO	11.7	0	88.3	14.3	0	85.7	12.9	0	87.1
CAZ	9.7	0	90.3	14.3	0	85.7	11.4	0	88.6
FOX	1.9	0	98.1	14.3	0	85.7	4.5	0	95.5
IPM	0	0	100	0	0	100	0	0	100
LVX	0	4.9	95.1	0	0	100	0	3.8	96.2
CSL	0	1.9	98.1	0	0	100	0	1.5	98.5

R: Resistant; I: Intermediate; S: Susceptible

[a]Total includes all *S.* 1, 4, [5], 12:i:- and *S.* Typhimurium strains, which are further divided into ST19, ST34, and a novel ST.
